# Comparative effectiveness of non-compounded polidocanol 1% endovenous microfoam (Varithena) ablation versus endovenous thermal ablation utilizing a systematic review and network meta-analysis

**DOI:** 10.1016/j.jvsv.2024.101896

**Published:** 2024-04-26

**Authors:** Lowell S. Kabnick, Juan Carlos Jimenez, Sheila M. Coogan, Larry Gache, Diana Frame, Candace Gunnarsson, Kathleen Ozsvath

**Affiliations:** a Vein and Lymphatic University, Lake Worth, FL; b Division of Vascular and Endovascular Surgery, David Geffen School of Medicine at University of California, Los Angeles, CA; c UT Health Houston, McGovern Medical School, Houston, TX; d LGache Statistical Consulting, Oakwood, OH; e Frame Research, Brooklyn, NY; f Gunnarsson Consulting, Jupiter, FL; g Samaritan Hospital, Troy, NY; h St. Peter’s Vascular Associates, St. Peter’s Medical Associates, Albany, NY

**Keywords:** Chronic venous insufficiency, Network meta-analysis, Polidocanol 1% endovenous microfoam, Varicose veins

## Abstract

**Objective:**

We compared the effectiveness and safety of polidocanol 1% endovenous microfoam ablation vs endovenous thermal ablation with radiofrequency or laser energy for treatment of venous insufficiency caused by lower extremity truncal vein incompetence via network meta-analysis of published comparative evidence.

**Methods:**

We conducted a systematic literature review following best practices, including a prospective protocol. We screened studies published in English from 2000 to 2023 for randomized and nonrandomized studies reporting direct or indirect comparisons between polidocanol 1% endovenous microfoam and endovenous thermal ablation. Thirteen studies met our eligibility criteria for the network meta-analysis. The co-primary effectiveness outcomes were the closure rate ≥3 months after procedure and the average change in the Venous Clinical Severity Score. For the subgroup of venous ulcer patients, the ulcer healing rate was the primary effectiveness outcome. The secondary outcomes included safety and patient-reported outcomes. Network meta-analyses were conducted on outcomes having sufficient data. Categorical outcomes were summarized using odds ratios (ORs) with 95% confidence intervals (CIs). Sensitivity tests and estimates of network inconsistency were used to investigate the robustness of our meta-analysis.

**Results:**

We found that polidocanol 1% endovenous microfoam was not significantly different statistically from endovenous thermal ablation for venous closure (OR, 0.65; 95% CI, 0.36-1.18; *P* = .16). Although not the primary aim of the study, the network meta-analysis also provided evidence to confirm our supposition that polidocanol 1% endovenous microfoam was significantly differentiated statistically from physician-compounded foam, with higher odds for vein closure (OR, 2.91; 95% CI, 1.58-5.37; *P* < .01). A sensitivity analysis using the longest available time point for closure in each study, with a minimum of 12 months of follow-up (median, 48 months; range, 12-72 months), showed results similar to those of the main analysis. No association was found between the risk of deep vein thrombosis and the treatment received. The available data were insufficient for a network meta-analysis of Venous Clinical Severity Score improvement and ulcer healing rates.

**Conclusions:**

Polidocanol 1% endovenous microfoam was not significantly different statistically from endovenous thermal ablation for venous closure and deep vein thrombosis risk for chronic venous insufficiency treatment, based on a network meta-analysis of published evidence. Polidocanol 1% endovenous microfoam was significantly differentiated statistically from physician-compounded foam, with higher odds of vein closure. A sensitivity analysis found venous closure findings were robust at follow-up intervals of 12 months or greater and for up to 6 years. New evidence meeting the inclusion criteria for this review will be incorporated at regular intervals into a living network meta-analysis.

## Rationale

Multiple treatment options are available for patients with symptomatic chronic venous insufficiency (CVI), a prevalent condition associated with varicose veins that occurs when venous insufficiency is present in the lower extremities' superficial and deep venous system. CVI can become clinically symptomatic, impacting activities of daily living and quality of life, and is often refractory to conservative therapy.^1^^,^^2^ The safety and efficacy of minimally invasive treatment options, including endovenous thermal ablation with radiofrequency or laser energy (ETA) and nonthermal techniques, have been studied extensively; however, published rates of technical success and symptom relief vary widely and are difficult to interpret.^3^

The utility of prior meta-analyses of CVI treatment options has been hampered by a failure to distinguish commercially available, Food and Drug Administration (FDA)-approved noncompounded polidocanol 1% endovenous microfoam ablation (Varithena; Boston Scientific) from other, nonstandardized foam sclerotherapy.^4^, ^5^, ^6^ Physician-compounded foam (PCF), using a liquid sclerosant manually combined with room air, filtered room air, or, less commonly, carbon dioxide, varies in composition and technique of application.^7^^,^^8^ In contrast, commercially manufactured polidocanol 1% endovenous microfoam (PEM) offers greater stability and cohesive properties in a biomimetic vein model compared with PCF, resulting in better overall performance.^8^ Additionally, chemical sclerosants (eg, polidocanol, sodium tetradecyl sulfate) and PEM differ in their FDA-approved prescribing information (labeled indications) and in the incidence of neurologic or cardiac adverse events reported to the FDA.^9^, ^10^, ^11^

At the time of the initial pivotal trials, PEM was not compared to ETA but to surgery, PCF sclerotherapy with the technique at the physician's discretion, or placebo.^12^, ^13^, ^14^, ^15^ More recently, nonrandomized studies have directly compared PEM and ETA.^16^^,^^17^ To incorporate all available comparative evidence, both randomized and nonrandomized studies comparing PEM and ETA, directly or indirectly, were included in our investigation.^18^ Given the breadth of the available evidence, we used a network meta-analysis approach to synthesize the literature on the effectiveness and safety of PEM and ETA treatments. A network meta-analysis pools information among treatments for a given medical condition, synthesizing evidence from both direct and indirect comparisons. The network approach allows us to investigate the two treatments using studies that directly compare PEM and ETA and studies that connect them through one or more common comparators. Combining both types of evidence statistically in a network meta-analysis improves estimation precision and, thus, provides more generalizable evidence on the relative effects of medical treatments.^18^, ^19^, ^20^

## Objectives

The objective of this study is to compare the effectiveness and safety of PEM ablation vs ETA in the primary treatment of adult patients with venous insufficiency caused by lower extremity truncal vein incompetence via a network meta-analysis of published comparative evidence (randomized or nonrandomized).

## Methods

### Protocol and registration

This systematic review was conducted under a prospective protocol, following current best practices for systematic reviews.^20^^,^^21^ The protocol is registered in PROSPERO (International Prospective Register of Systematic Reviews).^22^ The PRISMA (preferred reporting items for systematic reviews and meta-analyses) checklist extension for network meta-analysis has been completed and is on file with the journal.^23^ Studies published in English between January 2000 and January 2023 were searched using electronic databases and manual reference checks. Details of the search strategy are provided in Appendix A (online only). Two reviewers independently screened abstracts and, if potentially eligible, the full text for inclusion in the review; the study did not use automated screening algorithms. A standardized template was created for extracting relevant data for each included study (Appendix B, online only) and was completed independently by two reviewers. Any discrepancies in interpretation were resolved before the network meta-analysis, using a third reviewer if necessary. Additional data beyond what were available in the published report were sought for several studies; however, no responses were received. Thus, the analysis includes only published evidence.

### Eligibility criteria

Eligible studies were defined as CVI treatment studies with a randomized or nonrandomized comparison to at least one of the two treatments of interest (ie, PEM or ETA). We excluded single-arm studies, treatments not targeted to the truncal veins, combination treatments such as ETA plus phlebectomy, and studies without a common comparison of interest (ie, an alternate treatment used in at least one PEM study and one ETA study). The co-primary effectiveness outcomes were closure rate (occlusion) at time points of ≥3 months after the procedure and the mean or median change in the Venous Clinical Severity Score (VCSS, or its revised version, rVCSS). For the subgroup of venous ulcer patients (if reported), the primary effectiveness outcome was the venous ulcer healing rate. The secondary outcomes were safety (including total procedural complications, deep vein thrombosis [DVT], and any reported sequelae of thrombotic events) and patient-reported outcomes (including quality of life, symptom improvement, and patient preference). In general, outcomes were extracted as available from the included studies using the study authors' definitions of events. Thus, in studies that distinguished between DVT and endovenous heat-induced thrombus or endovenous foam-induced thrombus, only events described as DVT by the authors were extracted as DVT. A prospective statistical analysis plan specified that outcomes would be combined via meta-analysis, where sufficient and comparable data exist.

### Risk of bias assessment

Randomized trials were assessed for minimization of bias using the Jadad score, a 1 to 5 scale, with 5 indicating the highest quality.^24^ For comparative studies that were not randomized, we assessed how patients were allocated to treatment, whether studies were prospective or retrospective, and whether the authors described patients lost to follow-up. The presence of industry sponsorship was captured for eligible studies of any design.

### Planned methods of analysis

Network meta-analyses were conducted and reported following generally accepted best practices.^20^^,^^23^ We performed a frequentist meta-analysis using the R netmeta package, version 2.8-2.^25^ Categorical outcomes, including vein closure and DVT, were summarized with odds ratios (ORs) and 95% confidence intervals (CIs). For vein closure, we used an inverse variance weighting random effects model. For DVT, this study used the Mantel-Haenszel OR method with continuity correction due to the presence of zero events in a number of treatment groups. Continuous outcomes, such as the VCSS, were planned to be analyzed using the standardized mean difference comparing the change from baseline to follow-up between treatment groups. For all outcomes for which data permitted a meta-analysis to be conducted, two figures were created: (1) network geometry, with nodes denoting the included treatments and connecting lines representing the available pairwise comparisons, with the thickness of the lines showing the number of comparisons; and (2) a forest plot displaying the outcome estimates and corresponding 95% CIs for each pairwise comparison.

We evaluated network inconsistency—statistical disagreement between direct and indirect estimates—using local and global approaches appropriate to the analysis method.^26^^,^^27^ We assessed publication bias and other systematic heterogeneity related to sample size using comparison-adjusted funnel plots and Egger's regression test. Our analysis included sensitivity tests to investigate the robustness of the meta-analysis estimates. Venous closure outcomes were extracted at the time point closest to 12 months, which was used in the main analysis, and at the longest available time point (including in subsequent reports of the same study population), which was used in a sensitivity analysis. Studies reporting venous closure only at time points <12 months were excluded from the longest time point sensitivity analysis to ensure this analysis represented evidence on longer term closure.

## Results

**Fig 1:**
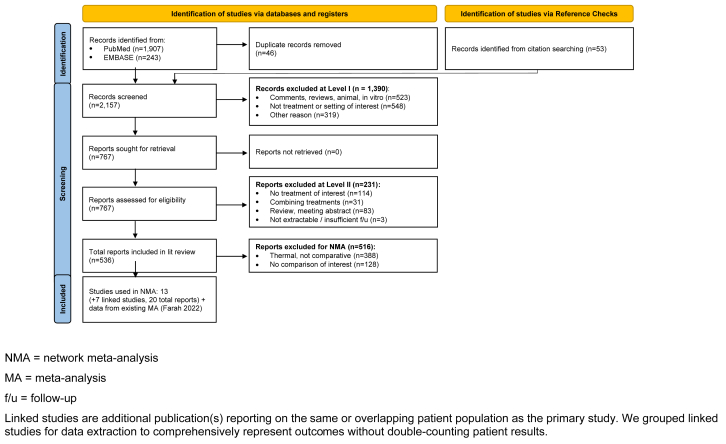
PRISMA (preferred reporting items for systematic reviews and meta-analyses) study attrition diagram.

A total of 2157 unique references were identified from all sources searched (Fig 1). Ultimately, 13 studies (20 total publications, due to multiple reports of the same or overlapping cohorts of patients) were eligible for inclusion in the network meta-analysis.^12^^,^^16^^,^^17^^,^^28^, ^29^, ^30^, ^31^, ^32^, ^33^, ^34^, ^35^, ^36^, ^37^, ^38^, ^39^, ^40^, ^41^, ^42^, ^43^, ^44^

### Treatment network

The majority of the articles screened did not report a comparison of interest. Common comparators between PEM and ETA were limited to surgery (primarily high ligation and stripping) and sclerotherapy with PCF. Our broad search did not identify any studies comparing PEM to cyanoacrylate adhesive or comparing ETA to placebo or sham procedures. Multiple studies, which varied in size, recency, and quality, compared ETA and surgery. A recent systematic review and meta-analysis (Farah et al,^45^ 2022) conducted for the 2022 Society for Vascular Surgery, American Venous Forum, and American Vein and Lymphatic Society guidelines was found in our search.^45^^,^^46^ Their meta-analysis featured similar eligibility criteria to this analysis (eg, inclusion of both randomized and nonrandomized comparative studies) and reported ETA vs surgery comparisons.^45^ To facilitate the network analysis and ensure alignment with the best evidence, we incorporated ETA vs surgery study data from the meta-analysis by Farah et al,^45^ using details available in the published appendix and omitting, where necessary, any duplicate studies that were also captured in our search.

### Study characteristics

Of the 13 included studies, comprising 233,801 patients, 6 were randomized controlled trials (RCTs), and 7 were comparative nonrandomized studies, either retrospective or prospective (Table I). Of the 13 studies, 4 included one or more PEM treatment groups. Our search yielded studies conducted primarily in the United States and Europe, with most patients enrolled in the United States. The range of CEAP (Clinical-Etiology-Anatomy-Pathophysiology) classification reported in the included studies was most often C2 to C6, with two RCTs (one PEM and one ETA) enrolling patients in class C2 to C4^12^^,^^39^ (Table II). The truncal veins treated were primarily the great saphenous vein, with two studies limited to patients with small saphenous vein incompetence.^35^^,^^42^ Concerning study quality, five of the six RCTs had a Jadad score of 3, reflecting proper randomization and reporting of withdrawals but no blinding to the treatment group. Most nonrandomized comparative studies were retrospective in nature and did not adjust for potential differences in patient characteristics between treatment groups. Three studies were industry-sponsored.

**Table I:** Summary of included studies

Variable	Studies, No.	Patients, No.
Total	13	233,801
Location		
North America	4	196,234
Europe	5	37,266
Asia	2	122
Other^a^	2	178
Industry sponsorship		
Yes	3	145,878
None reported	10	87,923
Study design		
RCT	6	2034
Comparative non-RCT^b^	7	231,767
Special patient population		
Female	1	80
Small saphenous vein	2	311

*RCT,* Randomized controlled trial.

a Egypt, Chile.

b Comparative non-RCTs could be prospective or retrospective and includes three large real-world data studies (Mallick et al,^36^ 2016; Pappas et al,^17^ 2021; and Sutton et al,^43^ 2012).

**Table II:** Study characteristics

Investigator	Study design; location	Study quality parameters	Comparisons	Patient population
Biemans et al,^28^^,^^29^ 2013	RCT; Netherlands	Jadad score 3; no industry sponsorship reported	ETA vs PCF; ETA vs surgery	n = 223; CEAP C2-C5; truncal veins: GSV; mean vein diameter, 6.1 mm
Brittenden et al,^30^, ^31^, ^32^, ^33^ 2014	RCT; United Kingdom	Jadad score 3; no industry sponsorship reported	ETA vs PCF; ETA vs surgery	n = 785; CEAP C2-C6; truncal veins: GSV and/or SSV; mean vein diameter, 8.7 mm GSV, 7.5 mm SSV
Deak,^16^ 2022	Comparative non-RCT; United States	Retrospective; industry sponsored; treatment allocation by patient choice and insurance coverage	PEM vs ETA	n = 1070; CEAP C2-C6; truncal veins: GSV and/or ASV; mean vein diameter, 7.9 mm
Gonzalez-Zeh et al,^34^ 2008	Comparative non-RCT; Chile	Prospective; no industry sponsorship reported; treatment allocation by patient choice	ETA vs PCF	n = 98; CEAP C2-C6; truncal veins: GSV (8.2% also perforators)
Hamel-Desnos et al,^35^ 2022	RCT; France	Jadad score 3; no industry sponsorship reported	ETA vs PCF	n = 161; CEAP C2-C5; truncal veins: SSV
Mallick et al,^36^ 2016; O'Donnell et al,^37^ 2015	Comparative non-RCT; United States	Retrospective; industry sponsored; treatment allocation: real-world data, not adjusted for confounders	ETA vs PCF; ETA vs surgery	n = 144,098; CEAP score not available; truncal veins not available
Mishra et al,^38^ 2016	RCT; India	Jadad score 3; no industry sponsorship reported	ETA vs PCF	n = 62; CEAP C2-C5; truncal veins: GSV
Mousa et al,^39^ 2019	RCT; Egypt	Jadad score 2; no industry sponsorship reported	ETA vs PCF; ETA vs surgery	n=80 (all female); CEAP C2-C4; truncal veins: GSV
Pappas et al,^17^^,^^40^^,^^41^ 2021	Comparative non-RCT; United States	Retrospective; no industry sponsorship reported; treatment allocation: real-world data, not adjusted for confounders	PEM vs ETA	n = 50,917; truncal veins: axial ablation groups without phlebectomy were extracted for both PEM and ETA; CEAP score not reported for this subset
Png et al,^42^ 2022	Comparative non-RCT; United States	Retrospective; no industry sponsorship reported; treatment allocation at provider discretion based on patient anatomy, allergies, preference, and insurance authorization	PEM vs ETA	n = 143; CEAP C2-C6; truncal veins: SSV
Sutton et al,^43^ 2012	Comparative non-RCT; United Kingdom	Retrospective; no industry sponsorship reported; treatment allocation: real-world data, not adjusted for confounders	ETA vs PCF; ETA vs surgery	n = 41,801; CEAP score not available; truncal veins: GSV and/or SSV, 30.8% not specified
Tiwary et al,^44^ 2020	Comparative non-RCT; India	Prospective; no industry sponsorship reported; treatment allocation not described	ETA vs PCF	n = 60; CEAP C2-C6; truncal veins: GSV
Wright et al,^12^ 2006	RCT; multinational (Europe)	Jadad score 3; industry sponsored	PEM vs PCF; PEM vs surgery (2 independently randomized cohorts)	n = 710; CEAP C2-C4; truncal veins: GSV and/or SSV

*ASV,* Anterior saphenous vein; *CEAP C,* Clinical-Etiology-Anatomy-Pathophysiology classification, clinical score; *ETA,* endovenous thermal ablation; *GSV,* great saphenous vein; *PCF,* physician-compounded foam; *PEM,* polidocanol 1% endovenous microfoam; *RCT,* randomized controlled trial; *SSV,* small saphenous vein.

### Synthesis of results

**Fig 2:**
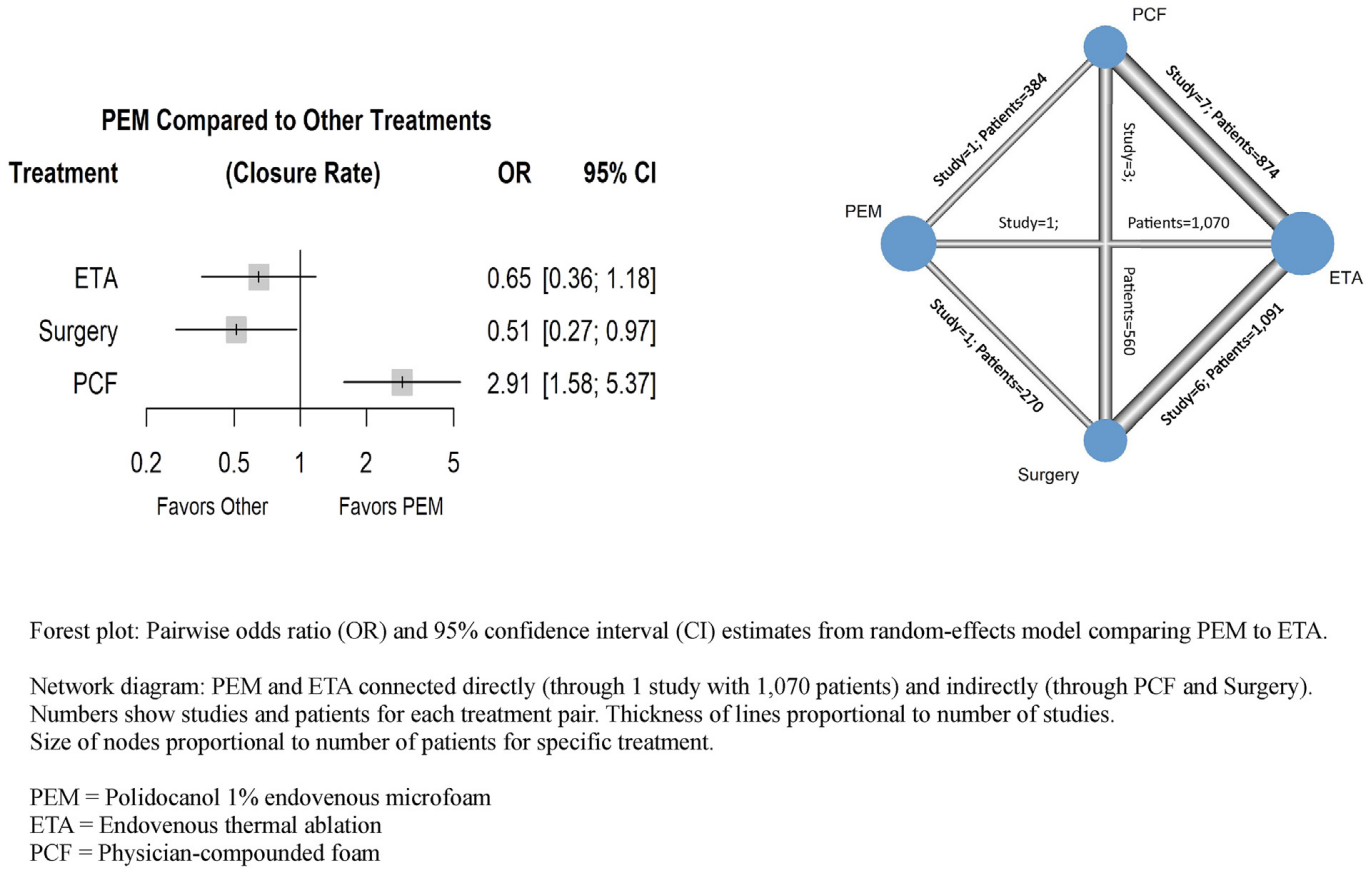
Network meta-analysis closure rate forest plot and network diagram.

Nine studies, supplemented by three ETA vs surgery studies from the Farah meta-analysis,^45^ supplied data on the primary end point of vein closure at a median time point of 12 months (range, 3-72 months). PEM was not significantly different statistically from ETA for vein closure (OR, 0.65; 95% CI, 0.36-1.18; *P* = .16). PEM was directly and indirectly connected to ETA in the network for this outcome, as shown in the network diagram (Fig 2). Although not the primary aim of the study, the network meta-analysis also provided evidence to confirm our supposition that PEM was significantly differentiated statistically from PCF, with higher odds for vein closure (OR, 2.91; 95% CI, 1.58-5.37; *P* < .01). A sensitivity analysis using the longest available time point for closure in each study, with a minimum of 12 months of follow-up (median, 48 months; range, 12-72 months), showed results similar to the main analysis for both ETA (OR, 0.69; 95% CI, 0.38-1.24; *P* = .21) and PCF (OR, 4.06; 95% CI, 2.19-7.53; *P* < .01). There was no evidence of publication bias or small-study effects (funnel plots and Egger's regression test results are presented in Appendix C, online only). Tests on the assumption of network consistency found little to no evidence of local or global network inconsistency (network *I*^*2*^ = 38.7%; 95% CI, 0.0%-67.5%).

**Fig 3:**
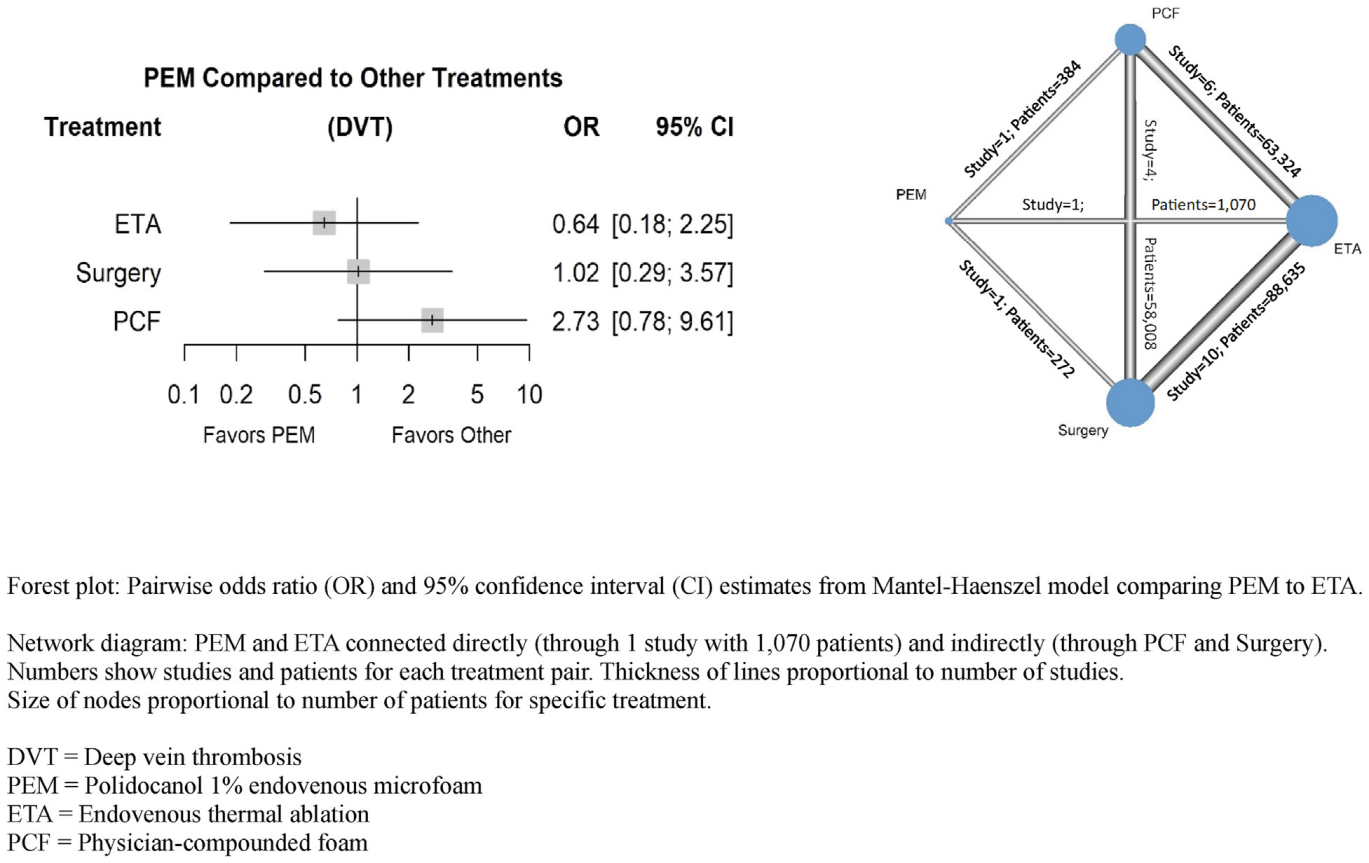
Network meta-analysis deep vein thrombosis (DVT) forest plot and network diagram.

Safety outcomes were inconsistently reported and could be analyzed only when the definition of total events was similar across studies or when sufficient studies reported the same specific event. Most studies reporting the total number of periprocedural complications did not specify the definition of these events or reported selected specific events without stating whether those were the sole events. Postprocedural DVT was, therefore, the only safety outcome meeting criteria for meta-analysis. Of the 13 studies, 12, plus 6 ETA vs surgery studies from the Farah meta-analysis,^45^ supplied data on DVT. There is no evidence that PEM is associated with an increased risk of DVT compared with ETA, surgery, or PCF treatment (Fig 3). Most DVTs were subclinical and asymptomatic, and sequelae such as pulmonary embolism were rare; asymptomatic DVTs were generally found on Doppler ultrasound scan.^16^^,^^34^^,^^42^ The literature did not commonly report details on the definition and timing of thrombotic events. Testing for global inconsistency was not possible for this outcome: a network *I*^*2*^ calculation and global decomposition of designs cannot be implemented for the Mantel-Haenszel method, which was chosen due to the presence of zero events in a number of treatment groups. As such, local network inconsistency tests were performed, and no evidence of network inconsistency was found. Some evidence of small study effects was found in the comparison-adjusted funnel plot and regression test (Appendix C, online only).

Within the included studies, certain outcomes we planned to summarize had insufficient data to be reliably included in the network meta-analysis: VCSS/rVCSS, venous ulcer healing, total procedural complications, and patient-reported outcomes. Six studies, plus eight from the Farah meta-analysis,^45^ supplied data on the VCSS/rVCSS change at 3 to 12 months after the procedure. Despite all groups showing improvements in the mean postprocedure VCSS/rVCSS, a meta-analysis of VCSS improvement could not be reliably performed due to limited evidence on the number of patients assessed, required imputation of means and standard deviations, and undue reliance on a single study intended to assess differences between patient age groups (<65 vs ≥65 years), not treatment.^17^ Similarly, the data were insufficient for the subset of patients with venous ulcers at baseline to conduct a meta-analysis on the ulcer healing rate. Patient-reported outcomes such as quality of life and postoperative pain were also not analyzed due to limited comparative data and the variety of scales used. As with the VCSS, studies reported significant improvement in quality of life after CVI treatment regardless of the treatment modality, with improvement observed even in cases with persistent reflux.^17^^,^^28^

## Discussion

We based this network meta-analysis on a thorough systematic literature review of English-language publications through January 2023. The analysis first aimed to characterize PEM evidence separately from evidence related to other, more commonly studied foams and to compare PEM to ETA via both direct and indirect comparisons. Treatment groups for surgery and PCF were used as common comparators in the network.

We found no statistically significant difference in vein closure rates between PEM and ETA; however, PEM was statistically significantly differentiated from PCF, with higher odds of vein closure. The analysis also showed no difference in the rates of postprocedure DVT between PEM and ETA. In fact, the 95% CIs were wide, and no statistically significant difference was seen between PEM and any of the treatment modalities in the network (ETA, surgery, PCF). For VCSS improvement, a meta-analysis could not to be conducted with the current evidence. Additional comparative evidence on patient-reported outcomes in this setting is also needed. For the purposes of a meta-analysis, improved reporting of such details as the number of patients administered follow-up scales would facilitate future assessments of the impact of CVI treatments on patients.

### Strengths and limitations

The strengths of this systematic review and network meta-analysis include the broad literature search incorporating electronic databases and manual reference checks, adherence to best practices including a prospective protocol, and rigorous network meta-analytic techniques. The limitations consist of those typical for meta-analyses based on existing evidence. Insufficient data were available for some outcomes, and treatment techniques and definitions of events could have varied by study author. Nonrandomized studies were not adjusted for patient characteristics at baseline, and neither randomized nor nonrandomized studies blinded patients to the treatment received, which was likely impractical. Some evidence of small study effects was seen for the DVT analysis, as was also the case in a previous meta-analysis of thermal ablation.^47^ This could indicate that DVT rates are underreported in small studies (publication bias) and/or that definitions of what constitutes a DVT, vs endovenous heat-induced thrombus or endovenous foam-induced thrombus, are evolving and inconsistently applied.^48^ The 95% CIs for some outcomes are wide, and tests of network inconsistency had low power due to the small number of studies in the network. When additional publications are available comparing PEM to other treatments, an update of the network meta-analysis should generate more precise pairwise comparisons and might allow for subanalyses by important patient characteristics. Studies published after our search cutoff date might contribute relevant comparative data.^49^^,^^50^ A prospective plan is in place to incorporate new evidence into a living meta-analysis.^51^

## Conclusions

PEM was not significantly different statistically from ETA for vein closure and DVT risk for chronic venous insufficiency treatment. This network meta-analysis of published evidence confirmed our supposition that PEM was significantly differentiated statistically from PCF, with higher odds for vein closure. A sensitivity analysis found venous closure outcomes were robust at follow-up intervals of 12 months or greater and up to 6 years. New evidence will be incorporated into the living network meta-analysis periodically (available at: https://www.varithena.com/en-us-hcp/clinical-evidence/living-meta-analysis.html).

## Author contributions

Conception and design: LK, LG, DF, CG

Analysis and interpretation: LK, JJ, SC, LG, DF, CG, KO

Data collection: LG, DF, CG

Writing the article: LK, LG, DF, CG

Critical revision of the article: LK, JJ, SC, LG, DF, CG, KO

Final approval of the article: LK, JJ, SC, LG, DF, CG, KO

Statistical analysis: LG, DF, CG

Obtained funding: Not applicable

Overall responsibility: LK

## Disclosures

L.S.K. provides education and consulting services to Boston Scientific, Beck and Dickinson, Amsel Medical, and AngioDynamics. J.C.J. is a consultant and speaker for Boston Scientific. S.M.C., L.G., D.F., and C.G. are consultants for Boston Scientific. K.O. is a speaker/advisory consultant for Boston Scientific, Medtronic, Terumo and Convatec; primary investigator for the W.L. Gore & Associates Venous stent trial; primary investigator for SAAVE study enVVeno Medical Corporation, serves on the Intersocietal Accreditation Commission Vascular Interventional Board; and is a Capital District Physicians’ Health Plan committee member.
