# Supplementary material for: Comparative effectiveness of non-compounded polidocanol 1% endovenous microfoam (Varithena) ablation versus endovenous thermal ablation utilizing a systematic review and network meta-analysis
Source: J Vasc Surg Venous Lymphat Disord. 2024 Apr 26;12(6):101896. doi: 10.1016/j.jvsv.2024.101896 (PMC11523335; doi:10.1016/j.jvsv.2024.101896)
Supplement: Supplementary Material [file mmc1.docx]

**Supplemental Information**

1. **Search Method Details**

Studies published in English language peer-reviewed journals between January 1, 2000 and January 31, 2023 were eligible for inclusion. The National Library of Medicine's PubMed database was the primary source for the electronic search. The Excerpta Medica (EMBASE®) database from Elsevier B.V., the Cochrane Library CENTRAL register, and manual reference checks supplemented the PubMed search.

We searched the PubMed web interface (https://pubmed.ncbi.nlm.nih.gov/) using the following terms, where "MeSH" indicates a MEDLINE Medical Subject Heading, and "NOT medline[sb]" indicates keywords to be sought in records not yet indexed by MEDLINE:

1. Varicose Veins/therapy[MeSH] OR Venous Insufficiency/therapy[MeSH] OR Varicose Veins/surgery[MeSH] OR Venous Insufficiency/surgery[MeSH] OR Saphenous Vein/surgery[MeSH]

2. ("varicose veins" OR "chronic venous insufficiency" OR “superficial venous insufficiency” OR "Saphenofemoral Junction Incompetence" OR "Saphenous Vein" OR "GSV incompetence") NOT medline[sb]

3. "radiofrequency ablation" OR "RF ablation" OR "laser ablation" OR "laser coagulation" OR EVLA OR EVLT OR "endovenous laser" OR "thermal ablation" OR ("great saphenous vein" AND ablation) OR polidocanol OR microfoam OR Varithena OR Varisolve OR "foam sclerotherapy" OR sclerosant OR ("non-thermal" AND "non-tumescent")

4. (#1 OR #2) AND #3; Limits: English, 2000-2023

EMBASE was searched for non-duplicative citations using search terms similar to the above, with Emtree® terms used in place of MeSH terms. We searched the Cochrane CENTRAL database for non-duplicative citations of potentially eligible comparative studies and recent systematic reviews of varicose vein treatment. Manual review of bibliographic references on studies eligible for data extraction and prior systematic reviews (past 5 years) were conducted to identify any potentially eligible studies missed during the electronic searches. The search cut-off date was January 31, 2023; however, articles made available online before being indexed in a journal (Epub ahead of print) were eligible for inclusion as long as the date of electronic publication was before the search cut-off date.

1. **Data Elements Sought in Eligible Studies**

Study Characteristics

- PMID (unique PubMed identifier)
- First author, Year published
- Title, Journal, Volume: Pages
- Location (Country or countries in which patients were treated, or Multinational if more than 3 countries)
- Study design: Randomized controlled trial (RCT); split-body or within-patient RCT; Comparative non-RCT; Prospective single-arm; Retrospective case series
- Intervention(s) studied
- Comparison (Varithena / thermal ablation vs. _________)
- Total number of patients in study
- Median follow-up (months)
- Linked studies (list publications with the same or overlapping populations)

Patient Characteristics (for each treatment group)

- Number of patients in treatment group
- Number of legs treated
- Intervention category (Varithena, Other foam, Thermal ablation, Surgery, No treatment)
- Intervention details (sclerosant formulation, type of ablation or surgery, administration, volume of foam)
- Clinical severity: Number of patients by strata (C2-C6)
- Age (mean/median, range)
- Number of males / females
- Race / ethnicity
- Body mass index (BMI): Mean/median, or N in categories as classified by authors
- Venous thromboembolism history (number of patients)
- Anti-coagulation therapy (number of patients)
- Vein diameter (mm): Mean/median and range of veins treated, or N in categories as classified by authors
- Number with deep venous reflux at baseline

Outcomes (for each treatment group)

Extract categorical outcomes with numerator and denominator (n/N) on intent-to-treat basis, if available. Extract mean and SD (preferentially) or median and range (or intraquartile range, IQR) for continuous variables. Capture time period of outcome assessment; for multiple timepoints, capture latest reported and 12-month outcomes; note availability of other timepoints.

- Effectiveness outcomes
  - Closure rate (%, n/N with complete occlusion) and timepoint(s) of assessment
  - Venous Clinical Severity Score (VCSS)
  - Heaviness, Achiness, Swelling, Throbbing, Itching (HASTI) score
  - Ulcer healing (for patients with venous ulcers/C6 at baseline)
- Safety outcomes
  - Procedure complications
  - Deep vein thrombosis (DVT)
    - Note if active monitoring (post-operative DVT monitoring with subclinical events included in total)
    - Note if available by location (e.g. below knee / above knee / remote DVT / proximal DVT extension)
  - Other adverse events (e.g. thrombophlebitis, pigmentation, pain, neurological events)
- Patient-reported outcomes
  - Post-operative pain
  - Symptom improvement
  - Patient preference
  - Appearance
  - Tolerability
  - Quality of life: list scale(s), e.g. Chronic Venous Insufficiency Quality of Life Questionnaire (CIVIQ), Aberdeen Varicose Vein Questionnaire (AVVQ)
- Notes on outcomes
- Outcomes for predefined subgroups:
  - Patients with venous ulcers (C6)
  - Vein characteristics at baseline: location (e.g. patients treated for SSV incompetence), reflux, vein diameter categories (e.g. >10 mm or <=10 mm)
  - Elderly
  - Obesity / BMI categories

Study Risk of Bias Assessment

Each included study will be appraised for methodologic quality by two independent reviewers using the Jadad scale for RCTs (Jadad 1996) and the following parameters for non-randomized comparative studies:

- Patient allocation to treatment
- Notes on patients lost to follow-up
- Primary endpoint pre-specified? Yes / no (for prospective non-randomized studies)
- Adequately powered? Yes / no (for prospective non-randomized studies)
- Industry sponsorship, based on author disclosures or affiliation

1. **Study Data Listings and Funnel Plots for Meta-analyses**

Study Listing for Vein Closure

| **Study** | **Treatment Arm** | **Group Size** | **Closure** | **Rate** | **Timing (Months)** |
| --- | --- | --- | --- | --- | --- |
| Biemans 2013 | B | 78 | 69 | 88.5% | 12^ |
| Biemans 2013 | C | 68 | 60 | 88.2% | 12^ |
| Biemans 2013 | D | 77 | 56 | 72.7% | 12^ |
| Brittenden 2014 | B | 141 | 116 | 82.3% | 6 |
| Brittenden 2014 | C | 173 | 135 | 78.0% | 6 |
| Brittenden 2014 | D | 182 | 79 | 43.4% | 6 |
| Deak 2022 | A | 550 | 514 | 93.5% | 43 |
| Deak 2022 | B | 520 | 482 | 92.7% | 43 |
| Gonzalez-Zeh 2008 | B | 45 | 42 | 93.3% | 12 |
| Gonzalez-Zeh 2008 | D | 53 | 41 | 77.4% | 12 |
| Hamel-Desnos 2022 | B | 65 | 60 | 92.3% | 12^ |
| Hamel-Desnos 2022 | D | 67 | 45 | 67.2% | 12^ |
| Mishra 2016 | B | 31 | 31 | 100.0% | 3 |
| Mishra 2016 | D | 30 | 28 | 93.3% | 3 |
| Mousa 2019 | B | 20 | 18 | 90.0% | 72 |
| Mousa 2019 | C | 35 | 32 | 91.4% | 72 |
| Mousa 2019 | D | 25 | 15 | 60.0% | 72 |
| Tiwary 2020 | B | 30 | 29 | 96.7% | 12 |
| Tiwary 2020 | D | 30 | 15 | 50.0% | 12 |
| Wright 2006 (Sclerotherapy) | A | 259 | 232 | 89.6% | 12 |
| Wright 2006 (Sclerotherapy) | D | 125 | 95 | 76.0% | 12 |
| Wright 2006 (Surgery) | A | 176 | 111 | 63.1% | 12 |
| Wright 2006 (Surgery) | C | 94 | 81 | 86.2% | 12 |
| Ay 2021 ^1^ | B | 70 | 61 | 87.1% | 12 |
| Ay 2021 | C | 62 | 62 | 100.0% | 12 |
| Rasmussen 2011 ^1^ | B | 213 | 200 | 93.9% | 12 |
| Rasmussen 2011 | C | 97 | 93 | 95.9% | 12 |
| Venermo 2016 ^1^ | B | 73 | 71 | 97.3% | 12^ |
| Venermo 2016 | C | 61 | 59 | 96.7% | 12^ |

A = PEM; B = ETA; C = Surgery; D = PCF

^1^ Study from Farah, *et al.* 2022 systematic review and meta-analysis

^ Longer follow-up data also available within publication (Hamel-Desnos 2022) or in a separate report (VanDerVelden 2015, Vahaaho 2018)

Vein Closure Comparison-adjusted Funnel Plot and Regression Test


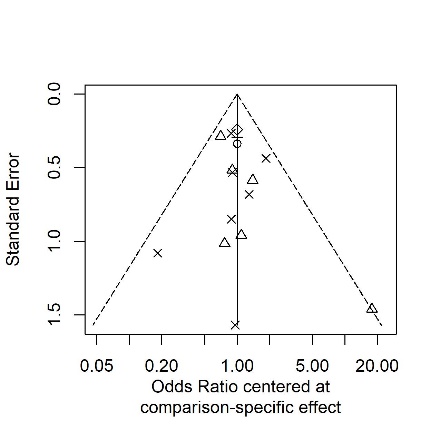


Egger's regression test p-value = 0.4596. No evidence of publication bias or small-study effects.

Study Listing for DVT

| **Study** | **Treatment Arm** | **Group Size** | **DVT** | **Rate per**  **100 patients** | **Timing**  **(Months)** |
| --- | --- | --- | --- | --- | --- |
| Biemans 2013 | B | 78 | 0 | 0.00 | 3 |
| Biemans 2013 | C | 68 | 0 | 0.00 | 3 |
| Biemans 2013 | D | 77 | 0 | 0.00 | 3 |
| Brittenden 2014 | B | 210 | 0 | 0.00 | 6 |
| Brittenden 2014 | C | 289 | 0 | 0.00 | 6 |
| Brittenden 2014 | D | 286 | 3 | 1.05 | 6 |
| Deak 2022 | A | 550 | 2 | 0.36 | 1 |
| Deak 2022 | B | 520 | 4 | 0.77 | 1 |
| Gonzalez-Zeh 2008 | B | 45 | 0 | 0.00 | Post-procedural |
| Gonzalez-Zeh 2008 | D | 53 | 2 | 3.77 | Post-procedural |
| Hamel-Desnos 2022 | B | 78 | 2 | 2.56 | 1 |
| Hamel-Desnos 2022 | D | 82 | 7 | 8.54 | 1 |
| Mallick 2016 | B | 44,617 | 1,655 | 3.71 | 1 |
| Mallick 2016 | C | 11,529 | 277 | 2.40 | 1 |
| Mallick 2016 | D | 12,708 | 104 | 0.82 | 1 |
| Mishra 2016 | B | 31 | 0 | 0.00 | NR |
| Mishra 2016 | D | 30 | 0 | 0.00 | NR |
| Mousa 2019 | B | 20 | 0 | 0.00 | NR |
| Mousa 2019 | C | 35 | 0 | 0.00 | NR |
| Mousa 2019 | D | 25 | 1 | 4.00 | NR |
| Png 2022 | A | 37 | 0 | 0.00 | 6 |
| Png 2022 | B | 113 | 0 | 0.00 | 6 |
| Sutton 2012 | B | 1,499 | 6 | 0.40 | 12 |
| Sutton 2012 | C | 29,435 | 108 | 0.37 | 12 |
| Sutton 2012 | D | 3,701 | 5 | 0.14 | 12 |
| Tiwary 2020 | B | 30 | 0 | 0.00 | NR |
| Tiwary 2020 | D | 30 | 0 | 0.00 | NR |
| Wright 2006 (Sclerotherapy) | A | 259 | 2 | 0.77 | NR |
| Wright 2006 (Sclerotherapy) | D | 125 | 1 | 0.80 | NR |
| Wright 2006 (Surgery) | A | 178 | 9 | 5.06 | NR |
| Wright 2006 (Surgery) | C | 94 | 0 | 0.00 | NR |
| Ay 2021 ^1^ | B | 70 | 0 | 0.00 | Post-procedural |
| Ay 2021 | C | 62 | 1 | 1.61 | Post-procedural |
| Flessenkamper 2013 ^1^ | B | 142 | 1 | 0.70 | 2 |
| Flessenkamper 2013 | C | 159 | 1 | 0.63 | 2 |
| Gonzalez Canas 2021 ^1^ | B | 69 | 0 | 0.00 | NR |
| Gonzalez Canas 2021 | C | 70 | 1 | 1.43 | NR |
| Helmy ElKaffas 2011 ^1^ | B | 90 | 0 | 0.00 | Post-procedural |
| Helmy ElKaffas 2011 | C | 90 | 1 | 1.11 | Post-procedural |
| Liao 2021 ^1^ | B | 100 | 1 | 1.00 | NR |
| Liao 2021 | C | 100 | 1 | 1.00 | NR |
| Sincos 2019 ^1^ | B | 26 | 1 | 3.85 | NR |
| Sincos 2019 | C | 23 | 1 | 4.35 | NR |

A = PEM; B = ETA; C = Surgery; D = PCF

NR = not reported

^1^ Study from Farah, *et al.* 2022 systematic review and meta-analysis

DVT Comparison-adjusted Funnel Plot and Regression Test


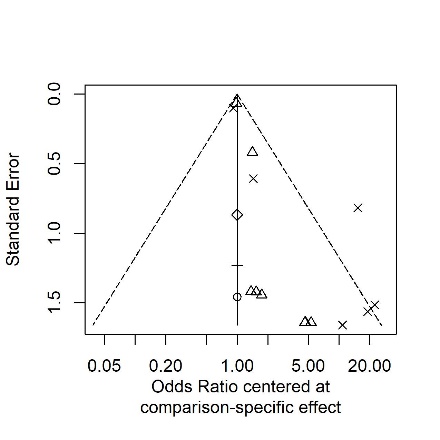


Egger's regression test p-value = 0.0015. Some evidence of publication bias or small-study effects.
